# SENSOR-ENHANCED CARE COORDINATION TO ADDRESS OLDER ADULT HEALTH: LESSONS FROM AN INTERDISCIPLINARY RESEARCH TEAM

**DOI:** 10.1093/geroni/igad104.0311

**Published:** 2023-12-21

**Authors:** Erin Robinson, Karla Washington

## Abstract

Researchers at the University of Missouri have a history of developing and testing aging in place (AIP) care coordination models for clinical translation. Vital to this work are in-home, residentially installed sensors to detect early illness and functional decline over time. While much smart home research is focused on older adults living in continuing care retirement communities, our team developed a novel sensor-enhanced care coordination approach for community-dwelling and disabled older adults as part of two ongoing, federally funded projects. For this symposium, we will highlight the Age-friendly Sustainable Smart and Equitable Technologies for Aging in Place (ASSETs for AIP) demonstration program, funded by the Department of Health and Human Services. Our first presentation will overview our interdisciplinary approach to creating this novel care coordination model, with specifics about research team composition, population focus, and methods. Our second presentation will highlight recruitment strategies and share lessons learned from reaching and enrolling this population that often experience residential transitions. Our third presentation will overview the open-source smart home and consumer-grade technologies deployed in this work, and practical considerations for technology installation, support, and sustainability and clinical team training. Our symposium will close with a presentation of selected client case studies that illustrate how clinical care coordinators use sensor data to remotely monitor and empower older adults for behavior change via telehealth consultations. This symposium will help inform other care coordination research, particularly that which uses sensor technologies and telehealth to facilitate aging in place.

